# Disaster preparedness knowledge and experiences among nurses during a competitive tabletop exercise program

**DOI:** 10.3389/fpubh.2026.1774598

**Published:** 2026-04-24

**Authors:** Bingbing Hu, Beibei Wang, Fanglin Li, Na Yang, Yuanyuan Chu, Shaohua Hu, Liangliang Sun

**Affiliations:** 1 Department of Neurosurgery, The First Affiliated Hospital of Anhui Medical University, Hefei, China; 2 Department of Nursing, The First Affiliated Hospital of Anhui Medical University, Hefei, China

**Keywords:** disaster preparedness, interdisciplinary collaboration, Kirkpatrick model, nurse, tabletop exercise, training

## Abstract

**Background:**

Disasters are increasing globally, necessitating enhanced disaster response competencies among frontline nurses. Tabletop exercise (TTX), as a low-resource, immersive scenario-based training tool, offers significant potential for improving nurses’ disaster preparedness. However, systematic evaluations of nurse-centered TTX programs remain scarce in China. This study aimed to comprehensively evaluate the effectiveness of a nurse-tailored competitive TTX program using the Kirkpatrick model.

**Methods:**

A sequential explanatory mixed-methods design was adopted. A 270-min TTX competition program covering three disaster themes was implemented for 90 nurses from 30 healthcare institutions in Anhui Province. In accordance with the Kirkpatrick model, quantitative data from 85 participants were collected using the Student Satisfaction and Self-Confidence in Learning Scale (SCLS), Simulation Design Scale (SDS), Nursing Disaster Emergency Knowledge Scale (NDEKS), and Educational Practices Questionnaire (EPQ). Additionally, a survey on disaster-related activities in participants’ institutions in 2025 was conducted. For the qualitative phase, maximum variation sampling was employed to purposely select 10 nurses for semi-structured interviews, and the qualitative data were analyzed using Colaizzi’s methodological framework.

**Results:**

The TTX program achieved positive outcomes across all four Kirkpatrick levels. At the Reaction Level, SCLS scores (4.42 ± 0.54) and SDS scores (4.38 ± 0.52) reflected nurses’ high satisfaction and recognition of program design. At the Learning Level, all six core domain scores of the NDEKS improved significantly post-TTX (all *p* < 0.001). At the Behavior Level, the EPQ total score reached 4.40 ± 0.55, indicating effective cultivation of positive behavioral tendencies. At the Results Level, the program drove remarkable regional disaster nursing development in Anhui Province in 2025, evidenced by over 220 science popularization activities, the establishment of new municipal committees, and diversified continuing education programs. Qualitative analysis identified four key themes, further supplementing and validating the quantitative findings of the TTX program’s multi-dimensional effectiveness.

**Conclusion:**

The nurse-tailored competitive TTX program, evaluated through the Kirkpatrick model, effectively enhances disaster knowledge, core competencies, and interdisciplinary collaboration while demonstrating tangible regional impact. This integrated evaluation framework provides robust evidence for optimizing disaster nursing training and strengthening healthcare system resilience.

## 1 Introduction

A disaster is an event caused by natural or human-made forces that threatens human lives and property, often requiring external assistance and resources to restore normalcy (1). Natural disasters typically include earthquakes, tornadoes, floods, tsunamis, and infectious disease outbreaks, while human-made disasters encompass incidents such as chemical spills, transportation accidents, and explosions (2). Globally, both the frequency and diversity of disasters are gradually increasing (3). For example, in 2021, 432 disaster events were recorded, resulting in 10,492 fatalities and affecting approximately 101.8 million people. Asia was particularly hard-hit, accounting for 40% of all disasters worldwide, 49% of total deaths, and 66% of the affected population (4). China, due to its large population, complex topography, and vast territory, is among the countries most frequently affected by disasters (5).

Nurses, as the largest group in the healthcare workforce, are indispensable across all four phases of disaster management: mitigation, preparedness, response, and recovery (6–8). Their competence in disaster response directly impacts the quality and efficiency of relief efforts, from triage and clinical care to resource coordination and psychological support for victims. Currently, compared with more developed countries, China’s disaster nursing education started late, educational content has not been unified, and disaster education and training has not been integrated into the basic nursing education curriculum (5). Related studies indicate that most nurses in China have not received systematic disaster training, lack rescue experience, and are not adequately prepared for disaster nursing (9–12). This deficiency hinders effective disaster response and underscores the urgent need for targeted training models.

The tabletop exercise (TTX), a scenario-based training method, has emerged as an effective tool for enhancing disaster preparedness (13–16). TTX is a low-resource, flexible simulation that uses scenarios, maps, and role-playing to guide participants in discussing and practicing disaster response protocols (17, 18). It has the potential to be comparatively less stressful for participants than high-fidelity simulation (HFS) or full-scale exercises (FSEs), yet can be equally immersive and as effective at bridging the gap between theoretical learning and practical application (19, 20). While TTX has been widely validated in healthcare training, few studies focus on nurses as core participants—especially in China, where context-specific, nurse-centered TTX programs aligned with local disaster risks (e.g., earthquakes, traffic accidents, infectious diseases) are scarce. Furthermore, existing evaluations of TTX often lack systematic frameworks, limiting the comprehensive capture of its effects at both the individual and organizational levels.

To address these gaps, this study developed a TTX competition program tailored for nurses, covering three typical disaster themes (serious traffic accidents, earthquakes, unexplained respiratory infectious diseases) and integrating interdisciplinary collaboration elements. Guided by Kirkpatrick’s Four-Level Training Evaluation Model—a globally recognized standard for assessing training effectiveness (21–23)—the study aimed to comprehensively evaluate the program’s impact across reaction, learning, behavior, and results dimensions. The specific objectives were to assess nurses’ satisfaction with the program (Reaction, Level 1), measure knowledge improvement (Learning, Level 2), explore nurses’ perceptions of educational practices that support behavior change as a process indicator for Level 3 (Behavior), investigate disaster-related activities organized by participants’ affiliated institutions in 2025 as an indicator of Level 4 (Results), and explore nurses’ subjective experiences through qualitative interviews, with the findings expected to provide evidence for optimizing disaster nursing training programs.

## 2 Methods

### 2.1 Design and evaluation framework

#### 2.1.1 Mixed-methods design

A sequential explanatory mixed-methods design was adopted, comprising two phases: (1) a quantitative phase, in which data were collected across the four levels of the Kirkpatrick model using validated instruments; and (2) a qualitative phase, designed to explore and contextualize the quantitative findings through in-depth semi-structured interviews. This design enabled quantitative data to provide initial evidence of the TTX program’s effectiveness, while qualitative data enriched understanding by capturing participants’ subjective experiences and contextual factors influencing outcomes (24).

#### 2.1.2 Evaluation framework: Kirkpatrick’s four-level training evaluation model

To ensure rigorous program evaluation, it is advisable to employ a validated evaluation framework incorporating evidence-based andragogical principles. A widely recognized and frequently applied tool is Kirkpatrick’s Four-Level Evaluation Model (21), which follows a four-tier sequential structure: Level 1 (Reaction), Level 2 (Learning), Level 3 (Behavior), and Level 4 (Results). Level 1 reflects participants’ satisfaction with and perceptions of the training program. Level 2 quantifies knowledge acquisition and skill improvement attributable to the intervention. Level 3 describes the extent to which training content is applied to job tasks and the degree to which participants’ professional roles are strengthened. Level 4 evaluates broader program outcomes, such as its overall influence within the professional domain (25, 26). This model enables assessment at both individual and organizational levels, providing reliable indicators of program efficacy and identifying areas for quality improvement (27). In this study, Level 3 was assessed using proximal indicators (participants’ perceptions of educational practices that facilitate behavior change), as direct observation of workplace behavior was not feasible in this immediate post-training evaluation.

### 2.2 Study population and samples

A total of 90 nurses from 30 healthcare institutions in Anhui Province participated in the disaster rescue TTX competition program (see Supplementary Appendix 1). To be eligible, nurses were required to meet three criteria: hold the position of nurse-in-charge, have at least 5 years of nursing experience, and provide informed consent to participate in the program. This study adhered to the ethical principles outlined in the Declaration of Helsinki, with ethical approval obtained from the Clinical Medical Research Ethics Committee of the First Affiliated Hospital of Anhui Medical University (approval no. PJ2025-06-93) on July 31, 2025. All study procedures complied with both national and international standards for research involving human participants.

The sample size was determined using G*Power 3.1.9.7 software based on *a priori* power analysis for paired samples *t*-test. Referring to the effect size of similar disaster training intervention studies, a medium effect size (*dz* = 0.4) was set (28). With a significance level (*α*) of 0.05 (two-tailed test) and a desired statistical power (1-*β*) of 0.90, the analysis indicated a minimum required sample size of 68 participants. Considering potential invalid questionnaires (e.g., missing data, logical inconsistencies) during data collection, an additional 20% sample margin was reserved to ensure sufficient statistical power. Consequently, the recruitment of 90 nurses from 30 healthcare institutions in Anhui Province met the study’s sample size requirements.

### 2.3 Intervention

#### 2.3.1 Development of TTX competition program

TTX aims to enhance preparedness for health emergency management through structured group discussions (16, 19). Consequently, its frameworks must be carefully designed to address specific challenges and achieve clearly defined objectives within predetermined timeframes (16, 29, 30). The effectiveness of a TTX program depends largely on its design and implementation (16, 29). To ensure high program quality, a design team of five researchers with extensive experience in emergency care education was established to oversee its development.

**Figure 1:**
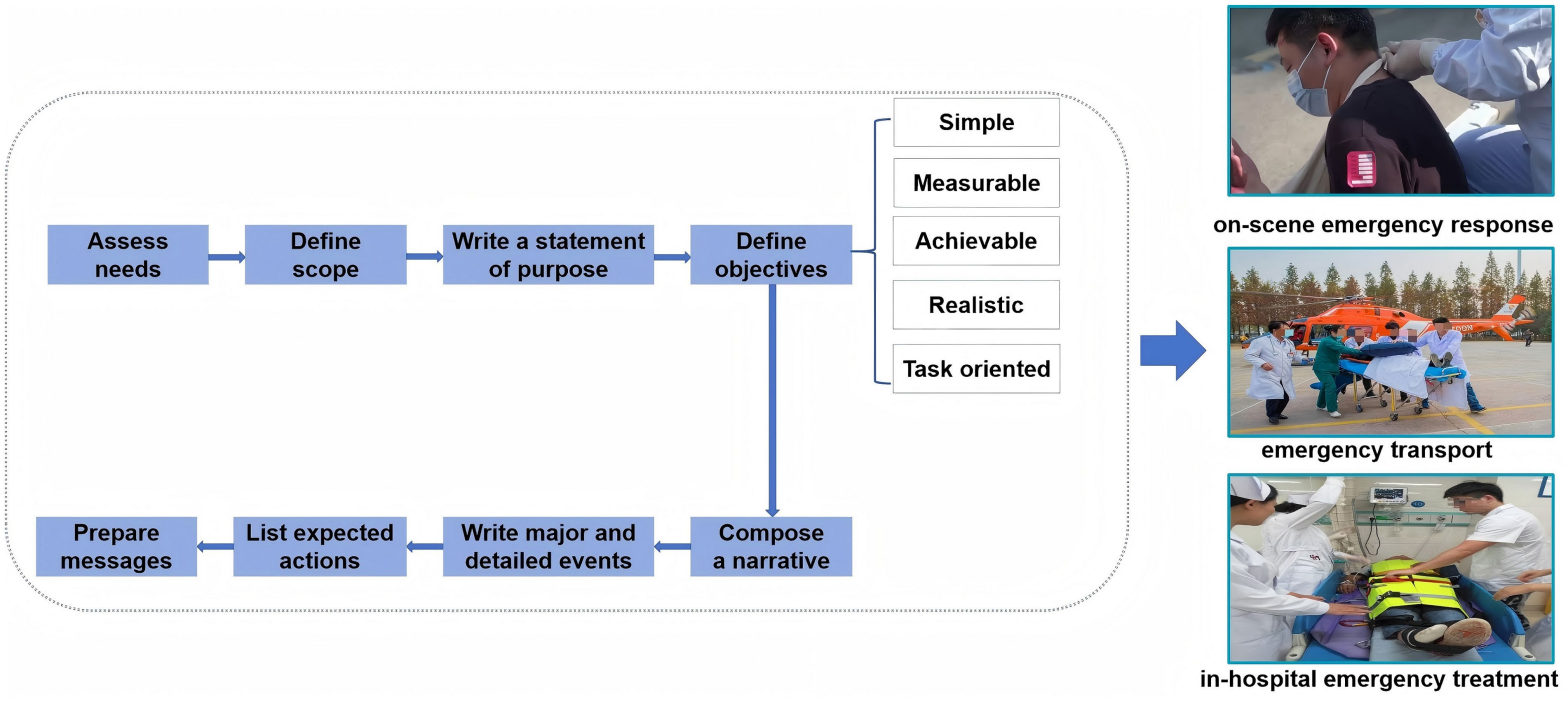
TTX program development process.

The development of the TTX program was based on the eight design steps for TTX proposed by the World Health Organization (29), encompassing three components: on-scene emergency response, emergency transport, and in-hospital emergency treatment (Figure 1). First, through a comprehensive needs assessment and multi-perspective analysis, three themes were identified for the TTX competition program: (1) earthquakes, (2) extraordinarily serious traffic accidents, and (3) unknown respiratory infectious diseases. A literature review was conducted to collect data on the progression and casualty figures of relevant typical emergencies, develop a concise incident timeline, and document the number of casualties as well as common injury types. Following the drafting of the TTX protocol, an expert meeting was convened to deliberate on the program’s objectives, content, format, and specific details. Additionally, the TTX program was developed based on the CO-S-TR model (16, 31, 32), incorporating C4 (Command, Control, Communication, Coordination), S4 (Staff, Stuff, Space, Special), and T4 (Tracking, Triage, Treatment, Transport). All 12 elements were fully integrated into the scenario (see Supplementary Appendix 2 for a detailed description of the CO-S-TR model and explanation of 12 elements). Researchers reviewed the audio recordings and on-site records of the expert conference, revised the preliminary TTX program, and finalized it. The core objectives of the TTX program are as follows: to improve and evaluate first aid strategies for sudden disaster events, requiring participants to select appropriate first aid techniques for each injury and attach triage cards to the injury models; to acquire and verify management competencies for sudden disasters; and to develop and validate assumptions and procedures related to emergency response plans. TTX scripts for extraordinarily serious traffic accidents are listed in Table 1.

**Table 1:** TTX script for extraordinarily serious traffic accidents.

Serial number	Information title	Event description	Technical points
1	A extraordinarily serious traffic accidents occured	At 2:00 p.m. on December 9, the emergency department of a tertiary hospital received an emergency call: A traffic accident occurred somewhere. A high-speed bus suddenly deviated from the lane and collided with a small car overtaking. The bus lost control and overturned instantly, rushing toward the right guardrail of the road. There were multiple casualties with varying degrees of injury inside and outside the vehicle.	Emergency start, activate response according to plan
2	Chaotic scene	Upon receiving the emergency call, medical personnel rushed to the scene of the accident. The vehicle was severely damaged, with large pools of blood visible on the road surface, along with scattered car glass and parts. Inside the vehicle, scattered personal belongings and food items could be seen. Firefighters and emergency medical personnel were assisting in transferring injured individuals from inside and outside the vehicle to a safe area.	On-site first aid and triage
3	Wound bleeding, forearm fracture	Injured person 1: hypersomnia, pale complexion, rapid breathing, R: 32 times/min, a 3 cm long wound on the inner side of the right forearm with jet-like bleeding. The right forearm is painful, deformed, and has limited mobility.	Hemostasis,bandaging, and fixation
4	Airway obstruction	Injured person 2: clearly conscious, in pain, right hand clutching neck in a “V” shape, face and lips purple, coughing violently, wheezing.	Diagnosis of airway obstruction by foreign bodies and the Heimlich maneuver
5	Cerebrospinal fluid leak	Injured person 3: a 5 cm x 5 cm hematoma on the forehead, with pale pink fluid dripping from the nasal cavity. Upon calling, the patient opened their eyes. During the medical history inquiry, the patient was incoherent, repeatedly stating “pain” and “headache.” When stimulated with pain, the limbs retracted. Sudden projectile vomiting occurred when transferring injured person from the car to a safe area.	Consciousness state assessment (GCS) and cerebrospinal fluid leakage management
6	Sudden decrease in blood oxygen saturation and cardiac arrest during on-site transportation	Injured person 4: an adult male, rescued from the vehicle and lying on the ground, clearly conscious, with swelling and bruising around the eyes and neck, bloody fluid visible from the right ear, swelling and deformity of the left lower leg, a 6 cm longitudinal laceration on the inner side of the left lower leg, bleeding has stopped, and the tibia is exposed. During transportation, the patient suddenly experienced a change in consciousness, was unresponsive to verbal stimuli, and exhibited a progressive decrease in blood oxygen saturation. Carotid artery pulses were absent.	Transportation precautions, tracheal intubation, and cardiopulmonary resuscitation
7	Hyperkalemia	Injured person 4: transported to the emergency department, biochemical test showed blood potassium level of 6.0 mmol/L and electrocardiogram showed an arrhythmia.	Emergency treatment for hyperkalemia
8	Open pneumothorax	Injured person 5: stabbed in the chest by a foreign object during a car accident, with clear consciousness. There is a 3 cm long wound on the chest, and the sound of air entering and exiting can be heard. Complaints: chest pain and difficulty breathing.	Open pneumothorax management and closed thoracic drainage

#### 2.3.2 Implementation of the TTX competition program

The disaster rescue TTX was implemented as a competitive team-based exercise, structured as a single-day 270-min training session with 90 min allocated to each of the three sequential scenarios: earthquakes, extraordinarily serious traffic accidents, and unexplained respiratory infectious diseases. The three scenarios were conducted consecutively (i.e., “back-to-back”) on the same day, with brief breaks scheduled between scenarios. All 90 participating nurses engaged in and completed all three scenario-based tabletop exercises.

The “competition” format was deliberately adopted to enhance engagement, motivation, and performance through inter-team rivalry. Participants were divided into multidisciplinary teams representing different healthcare institutions. Each team collaboratively responded to evolving scenarios, with performance evaluated by a panel of expert judges based on predefined criteria, including triage accuracy, clinical decision-making, teamwork effectiveness, and adherence to disaster management protocols. This competitive approach draws on established pedagogical principles: competition can increase intrinsic motivation, promote active learning, and foster accountability, thereby enhancing knowledge retention and skill acquisition (16, 30). Additionally, the competitive format creates a dynamic, time-sensitive learning environment that mirrors the intense pace of real disaster response operations, better preparing nurses for actual emergency scenarios.

Two experienced instructors facilitated the entire competition process. Prior to the exercise, a structured pre-briefing session was conducted to familiarize participants with the simulation objectives, the flow of activities, the use of instructional props, communication protocols, and the evaluation criteria, ensuring that all participants understood how the simulation would operate. During the exercise, the instructors’ responsibilities included: (1) introducing the scenario background and competition rules; (2) dynamically advancing the narrative based on participants’ responses and providing real-time updates on casualty conditions; (3) offering targeted feedback during the exercise; (4) leading structured post-exercise debriefing discussions to consolidate learning.

**Figure 2:**
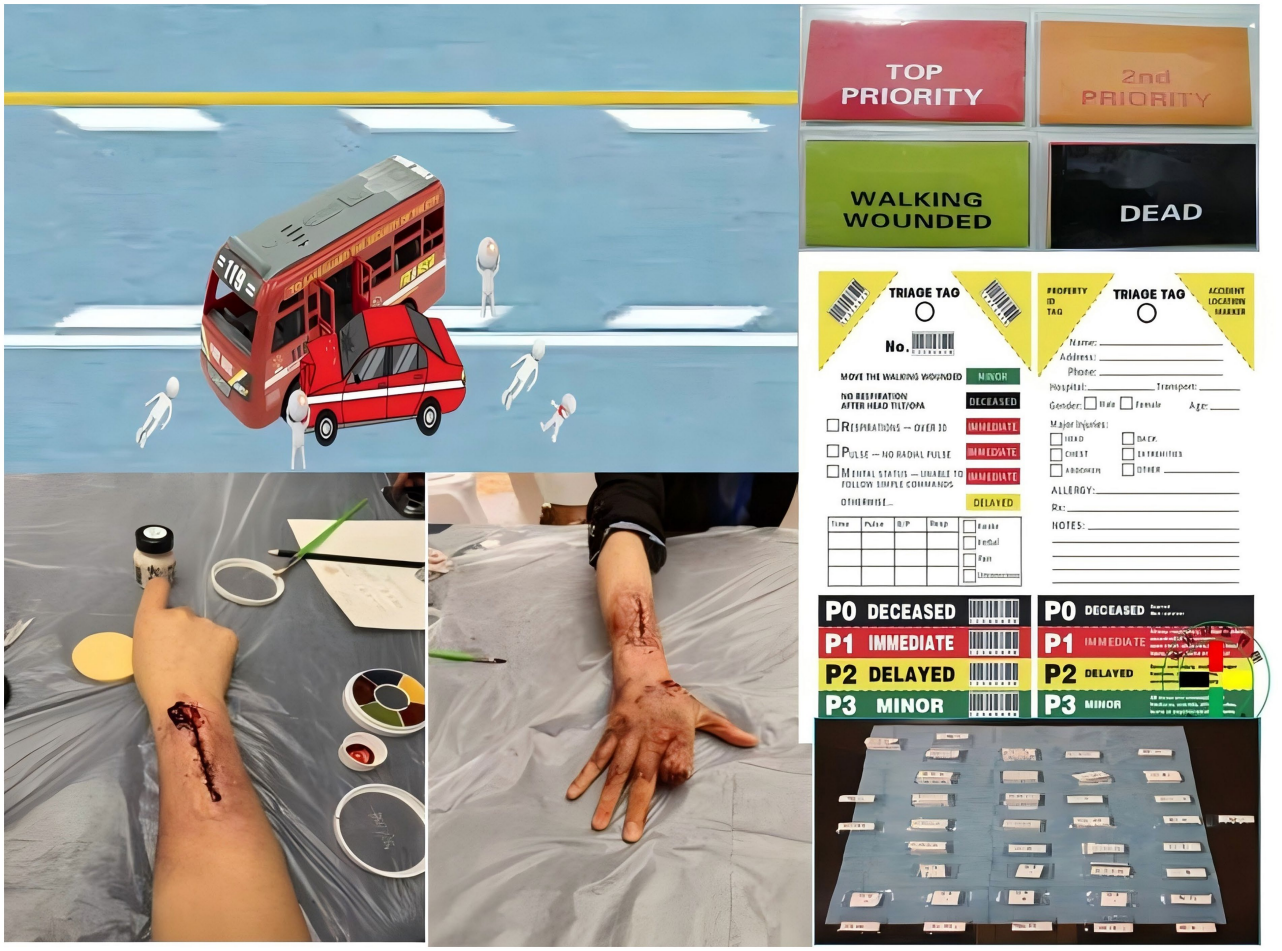
Instructional props.

To enhance the authenticity and effectiveness of the simulation, all instructional props (e.g., injury models, scenario maps, triage tags) were custom-designed to align with real disaster response scenarios (Figure 2). Additionally, instructors assumed auxiliary roles such as bystanders, media personnel, and injured patients to create an immersive practice environment. Each competitive round lasted 90 min.

### 2.4 Data collection

#### 2.4.1 Quantitative data collection

Cluster sampling was used to recruit participating nurses, and data were collected via self-administered questionnaires and organizational outcome investigations, with the four levels of the evaluation framework corresponding to specific measurement tools and data sources as follows: (1) Level 1 (Reaction) was assessed using the Student Satisfaction and Self-Confidence in Learning Scale (SCLS) and the Simulation Design Scale (SDS), both developed by the National League for Nursing (NLN). These instruments are widely used to evaluate novice nurses’ perceptions of simulation-based learning (33, 34). The Chinese versions translated and validated by Wang et al. (35) were employed in the present study, as their study confirmed satisfactory psychometric properties in terms of relevance and importance of the key scale dimensions. The 13-item SCLS measures nurses’ satisfaction with instructional design and self-confidence in simulation-based learning, while the 20-item SDS evaluates perceptions of core simulation elements including objectives and information, support, problem-solving, feedback, and fidelity. In this study, the SCLS and SDS demonstrated excellent internal consistency with Cronbach’s *α* coefficients of 0.978 and 0.982, respectively. All items were rated on a 5-point Likert scale (1 = strongly disagree, 2 = disagree, 3 = undecided, 4 = agree, 5 = strongly agree). (2) Level 2 (Learning) was measured by the Nursing Disaster Emergency Knowledge Scale (NDEKS) (10), which assesses mastery of disaster emergency knowledge across six core domains: incident command system in disaster care; triage; communication; special care, isolation and decontamination; reporting and access to essential resources; and biological preparedness (detailed descriptions of each domain are provided in Supplementary Appendix 3). The scale contains 40 items rated on a 5-point Likert scale (1 = absent, 5 = excellent), with total scores ranging from 40 to 200 (higher scores indicate greater disaster emergency knowledge). A pre-test was administered 1 week before the TTX and a post-test immediately after training completion; the scale had a Cronbach’s *α* coefficient of 0.987 in this study, indicating excellent internal consistency. (3) Level 3 (Behavior—Proximal Indicators) was evaluated using the Educational Practices Questionnaire (EPQ), a 16-item scale developed by the National League for Nursing, with the Chinese version validated by Wang et al. (35). This scale gauges participants’ perceptions of the presence and importance of educational best practices within simulation, including active learning, collaboration, learning diversity, and high expectation. The scale was rated on a 5-point Likert scale (1 = strongly disagree, 2 = disagree, 3 = undecided, 4 = agree, 5 = strongly agree) and had a Cronbach’s *α* coefficient of 0.983 in this study, reflecting good internal consistency. While EPQ does not directly measure post-training workplace behavior, it captures participants’ experiences of educational practices that are foundational to subsequent behavior change. (4) Level 4 (Results) was investigated through organizational outcome surveys of the 30 healthcare institutions with which the participants were affiliated, collecting objective data on disaster nursing-related activities organized by these units in 2025, including: (1) academic activities (national/provincial/municipal continuing education programs); (2) competitive events (emergency drill, TTX competitions, disaster nursing skill contests, and science popularization competitions); (3) science popularization activities (number of offline activities and innovative online popularization forms such as live streaming and short videos).

In addition, a demographic and work-related characteristics questionnaire was used to collect participants’ basic information, including gender, age, educational background, professional title, etc. All questionnaires were distributed with standardized instructions, and researchers provided guidance to ensure the completeness and validity of the collected data.

#### 2.4.2 Qualitative data collection

Qualitative research employed a combination of purposive and maximum variation sampling to select participating nurses as interview subjects. The sample size was determined by data saturation, considered achieved when no new themes emerged from subsequent interviews. Data were collected through individual semi-structured interviews conducted in Chinese. Using a digital voice recorder, researchers asked open-ended questions that were progressively deepened based on participants’ responses. The interview environment was relaxed, with researchers listening attentively without offering guidance. Each session lasted 30–40 min. During the interviews, interviewers closely observed participants’ facial expressions and emotional states while documenting their words, expressions, and movements in writing. After all interviews were completed, each participant was asked to review and confirm the written records to ensure accuracy. The semi-structured interview included open-ended questions such as: (1) What knowledge did you acquire or consolidate while preparing for the TTX competition program? (2) In which aspects did you improve through participation in the competition program? (3) Did the competition program help you acquire relevant knowledge? (4) What challenges did you encounter during the TTX competition program, and how did you address them? (5) Could you describe your overall psychological experience regarding the competition program? (6) What kind of disaster rescue training (e.g., TTX, HFS, FSE, or classroom lectures) would you prefer in the future? (7) Do you have any other suggestions for improving this TTX program? Interviews concluded when responses became repetitive, reaching a point known as “data saturation” where no new information emerged.

### 2.5 Data analysis

Data analysis was performed using IBM SPSS Statistics (Version 25.0). For the comparison of nurses’ disaster emergency knowledge scores before and after the TTX competition, a Wilcoxon signed-rank test (a paired sample nonparametric test) was employed to compare the median scores of the six core dimensions. Results were presented as median (interquartile range, IQR), and *Z* values with corresponding *p* values were reported to indicate statistical significance. A two-tailed *p* value < 0.05 was considered statistically significant for all quantitative analyses.

Qualitative data analysis followed Colaizzi’s methodological framework (36). A dual-researcher protocol ensured verbatim transcription of all interview recordings within 24 h, with supplementary observational notes on nonverbal behaviors integrated directly into the transcripts. Through iterative engagement with the textual data, researchers identified and extracted significant statements. These statements were discussed rigorously until consensus was reached on their formulated meanings. The team then organized the formulated meanings into a new table, grouping them into thematic clusters to identify common key concepts that served as prototypes for the emerging themes. These concepts were described in detail, with each theme defined using two or three representative statements. After compiling and comparing similar topics and descriptions to distill shared perspectives, each theme was summarized using concise phrases.

## 3 Results

### 3.1 Characteristics of participants

A total of 85 questionnaires were deemed valid, corresponding to an effective response rate of 94.44%. Among the 90 nurses who participated in the TTX program, two nurses declined to complete the questionnaire after the exercise, and three nurses had incomplete questionnaire information that rendered their responses invalid—these 5 cases were excluded from the final quantitative analysis. Of the valid respondents, 24 were male and 61 were female. Participants’ ages ranged from 26 to 48 years, with a mean of 33.60 ± 5.58 years. Their years of work experience averaged 11.74 ± 6.35 years, and 32 nurses had previously participated in rescue activities, as detailed in Table 2.

**Table 2:** Demographic information and work-related characteristics of participants in the disaster TTX competition program (*n* = 85).

Variables	n	%
Gender		
Male	24	28.24
Female	61	71.76
Age		
20–30 years	29	34.12
31–40 years	47	55.29
41–50 years	9	10.59
Educational level		
Below bachelor’s degree	37	43.53
Bachelor’s degree or above	48	56.47
Professional title		
Primary title	27	31.76
Intermediate title	52	61.18
Senior title	6	7.06
Work department		
Emergency department	60	70.59
Intensive care unit	9	10.59
Other departments	16	18.82
Work experience		
≤ 5 years	12	14.12
6–10 years	32	37.65
11–15 years	28	32.94
≥ 16 years	13	15.29
Hospital level		
Tertiary	75	88.24
Non Tertiary	10	11.76
Have attended disaster relief training		
Yes	35	41.18
No	50	58.82
Have participated in emergency drill for disaster event		
Yes	50	58.82
No	35	41.18
Have participated in rescue activities		
Yes	32	37.65
No	53	62.35

### 3.2 Level 1 (reaction): SCLS and SDS evaluation

Nurses reported high satisfaction with the TTX program and positively evaluated its simulation design. The mean score of the SCLS was 4.42 ± 0.54, with subscales of satisfaction with instruction (4.46 ± 0.52) and self-confidence with learning (4.40 ± 0.57). The SDS yielded a mean score of 4.38 ± 0.52, with the highest rating for feedback effectiveness (4.44 ± 0.57) and the lowest for support (4.33 ± 0.57). These high scores indicate that the TTX program effectively met nurses’ learning needs and demonstrated excellent design quality (Table 3).

**Table 3:** Scores for SCLS and SDS after the TTX competition (*n* = 85).

Scale	Dimension	No. of entry	Total scores	Mean scores
SCLS^1^	Satisfaction with instruction	5	22.32 ± 2.62	4.46 ± 0.52
	Self-confidence with learning	8	35.18 ± 4.53	4.40 ± 0.57
SDS^2^	Objectives and information	5	21.69 ± 2.96	4.34 ± 0.59
	Support	4	17.32 ± 2.28	4.33 ± 0.57
	Problem solving	5	22.01 ± 2.68	4.40 ± 0.54
	Feedback	4	17.75 ± 2.28	4.44 ± 0.57
	Fidelity	2	8.76 ± 1.02	4.38 ± 0.51

1 SCLS, Student satisfaction and self-confidence in learning scale.

2 SDS, Simulation design scale.

### 3.3 Level 2 (learning): NDEKS pre-test and post-test comparison

Nurses’ disaster emergency knowledge scores across six core dimensions were significantly improved after the TTX competition (all *p* < 0.001), as shown in Table 4. Triage showed the most significant improvement after the TTX competition, with the median score increasing from 3.60 (IQR = 0.50) to 4.00 (IQR = 0.90) (*Z* = 7.464, *p* < 0.001)—the highest *Z*-value among all dimensions, indicating the most notable enhancement. In contrast, biological preparedness had the least apparent improvement, and the median score remained unchanged at 3.00 (IQR = 1.00) both before and after the competition (*Z* = 5.281, *p* < 0.001). Although the difference was statistically significant, there was no quantitative increase in median score, making it the dimension with the minimal observable improvement.

**Table 4:** Comparison of NDEKS scores before and after the TTX competition (*n* = 85).

Dimension	Pre TTX competition median (IQR)^1^	Post TTX competition median (IQR)^1^	*Z*	*p*
Incident command system in disaster care	3.29(1.71)	3.86(0.86)	7.090	<0.001
Triage	3.60(0.50)	4.00(0.90)	7.464	<0.001
Communication	3.43(0.71)	3.86(1.07)	6.710	<0.001
Special care, isolation and decontamination	3.27(0.73)	3.47(1.00)	6.336	<0.001
Reporting and access to essential resources	3.67(0.83)	4.00(1.00)	3.664	<0.001
Biological preparedness	3.00(1.00)	3.00(1.00)	5.281	<0.001

1 IQR, Interquartile range.

### 3.4 Level 3 (behavior—proximal indicators): EPQ evaluation

The overall EPQ mean score was 4.40 ± 0.55, with all dimensions scoring above 4.0 (Table 5). Collaboration received the highest rating (4.47 ± 0.55), followed by learning diversity (4.46 ± 0.56), high expectation (4.44 ± 0.56), and active learning (4.37 ± 0.57). These results indicate that the TTX program effectively cultivated behavioral tendencies toward collaborative practice, proactive participation, and diverse learning approaches, which are foundational for workplace behavior change.

**Table 5:** Scores for EPQ after the TTX competition (*n* = 85).

Scale	Dimension	No. of entry	Total scores	Mean scores
EPQ^1^	Active learning	10	43.73 ± 7.74	4.37 ± 0.57
	Collaboration	2	8.94 ± 1.09	4.47 ± 0.55
	Learning diversity	2	8.92 ± 1.13	4.46 ± 0.56
	High expectation	2	8.88 ± 1.13	4.44 ± 0.56

1 EPQ, Educational practices questionnaire.

### 3.5 Level 4 (results): 2025 Anhui provincial disaster nursing development status

A survey of 30 participating healthcare institutions revealed extensive disaster nursing activities across Anhui Province in 2025. Academic activities included one national and five municipal continuing education programs. In terms of competitions, 12 institutions conducted 15 emergency drills, 3 institutions held TTX competitions, and 6 institutions organized science popularization contests. Science popularization efforts comprised over 220 offline activities reaching schools, communities, and enterprises, complemented by innovative online formats including live streaming and short videos—for instance, one hospital’s educational video attracted nearly 5,000 views. Additionally, committee members contributed to the publication of a disaster nursing textbook, and four new municipal disaster nursing committees were established, further strengthening the provincial disaster nursing network.

### 3.6 Qualitative findings on nurses’ training experiences in the TTX competition program

A total of 10 nurses were included as research subjects, comprising 3 males and 7 females. Their ages ranged from 29 to 48 years, and their years of work experience averaged 11.80 ± 7.22 years; 3 nurses had previously participated in disaster relief work. Through in-depth analysis and extraction of interview data, four themes were identified regarding nurses’ experiences of participating in emergency rescue TTX competition program for sudden disaster events, as summarized in Table 6.

**Table 6:** Training experiences of nurses who participated in TTX competition program (*n* = 10).

Themes	Description
Theme 1: Promoting the development of a structured disaster knowledge system	Nurse A: “As a nurse in the intensive care unit, I had not previously received formal training in trauma first aid and possessed only a rudimentary comprehension of triage. This TTX competition program enhanced my understanding by addressing my knowledge deficiencies.” Nurse D: “I did a lot of preparation work before the competition, systematically studied the recommended books for this TTX competition program, and searched for relevant disaster rescue materials online.” Nurse E:“After the competition program, I reviewed the entire knowledge framework and documented it in a memo.”
Theme 2: Enhancing critical thinking, teamwork, and communication skills	Nurse B: “TTX competition program allows me to systematically structure my knowledge, extract useful information, integrate it, broaden my thinking, and avoid unilateral thinking.” Nurse D: “Before the competition, we meticulously allocated the tasks and reviewed the key knowledge points. During the competition, we synthesized pertinent information and reached judgments via collaborative discussion.” Nurse F: “The atmosphere at the competition site was quite tense, so I had to concentrate fully and keep my mind clear.”
Theme 3: Simulating real disaster scenarios to increase immersion	Nurse D: “The TTX competition program provided real disaster scenarios and various unexpected situations, making it more realistic and challenging.” Nurse G: “This form is more impressive and systematic than traditional teaching, and through a whole set of deduction, the entire rescue procedure becomes clearer in the mind.” Nurse I: “I immersed myself in it, feeling like I was part of it all.”
Theme 4: Strengthening multidisciplinary collaboration	Nurse A: “The scenario of massive casualties is notably intricate. My prior involvement in emergency rescue has imparted the understanding that multidisciplinary knowledge is essential. TTX can facilitate our amalgamation of transdisciplinary knowledge.” Nurse J: “TTX allows team members to analyze problems from different disciplinary perspectives, making our analysis more comprehensive and detailed. In a sense, it is similar to a multidisciplinary case discussion.”

## 4 Discussion

### 4.1 TTX innovates a disaster training model, promoting the transition from theory to practice

Nurses constitute the largest workforce in healthcare and play a pivotal role in disaster response (37). Consequently, they must possess foundational knowledge and skills related to disaster care and be prepared to respond proactively to emergencies (38). Given the highly situational nature of disasters, traditional nursing education primarily emphasizes knowledge transmission, making it challenging to enhance practical disaster management competencies through applied learning. A key challenge in disaster nursing education is the gap between theoretical knowledge and practical application (39). With advances in educational and information technologies, TTX has been increasingly adopted to improve the effectiveness of disaster nursing education (16, 40, 41).

Exercises refer to focused practice that places participants in a simulated situation, where they are expected to act as they would during a real incident (29). There are five primary types of comprehensive exercises: orientation seminars, drills, TTX, functional exercises, and FSEs (29). TTX serves as a bridge between simplicity and complexity, narrowness and breadth, minimal and maximal implementation costs, and theoretical knowledge and practical application (19, 30). In TTX, participants assume designated roles to analyze and resolve problems based on existing operational plans, clarify coordination mechanisms and responsibility divisions, and engage in guided group discussions (19). These characteristics enable TTX to effectively enhance participants’ awareness of roles and responsibilities in disaster response, making them a widely adopted training method in emergency preparedness education (42, 43). Our program incorporated three typical disaster scenarios (serious traffic accidents, earthquakes, and unexplained respiratory infectious diseases) and used custom-designed instructional props to simulate real response contexts. This design not only reinforced theoretical knowledge such as triage and emergency treatment but also promoted its practical application, effectively addressing the theory-practice disconnect in traditional disaster nursing training.

### 4.2 The TTX competition solidifies foundational rescue knowledge and improves disaster response capabilities: a Kirkpatrick model analysis

Our study employed a validated evaluation framework, Kirkpatrick’s Four-Level Training Evaluation Model, which provides a systematic, stepwise approach to measure outcomes from participant reaction to organizational impact (21–23, 44). This model has been widely applied across healthcare education, from evaluating simulation-based training for skilled birth attendants in low-resource settings to assessing continuing education program for dental professionals and in-service training for nurses (27, 45, 46).

#### 4.2.1 Level 1 (reaction): high participant satisfaction and perceived educational value

The first level of the Kirkpatrick model measures participants’ reaction to the training. Our findings at this level indicated a high level of satisfaction and engagement. Nurses reported high satisfaction with the TTX program, as evidenced by the high mean scores on the SCLS, particularly in satisfaction with instruction. The positive evaluation of the simulation design using the SDS further confirms that the program’s structure, objectives, and fidelity effectively met learners’ needs.

This high level of satisfaction and positive evaluation can be largely attributed to the immersive experience provided by the TTX program, which was fully reflected in our qualitative findings (Theme 3: simulating real disaster scenarios to increase immersion). Participants consistently reported that the custom-designed props, dynamically progressing scenarios, and immersive simulations created an authentic atmosphere. This finding aligns with Liu et al. (16), who demonstrated that mid-fidelity, real incident-based TTX scenarios significantly increased participant involvement, as well as with Sena et al. (43), who showed that TTX-based disaster training was well-received and significantly improved learners’ self-reported confidence in disaster response.

Notably, while the “support” subscale on the SDS received a slightly lower score (4.33 ± 0.57), it remained relatively high. This suggests that some participants may have felt somewhat uncertain about how to effectively utilize available resources and information when first encountering this novel TTX format. This finding provides direction for our future work, highlighting the need to strengthen pre-exercise orientation and provide more timely support during the process to help participants more quickly adapt to and fully leverage the TTX environment.

#### 4.2.2 Level 2 (learning): significant and multi-dimensional knowledge acquisition

Level 2 assesses actual learning through increases in knowledge, skills, or attitude changes. Our quantitative results robustly demonstrate TTX effectiveness at this level, with significant improvements across all six NDEKS dimensions (all *p* < 0.001). These findings align with studies documenting learning gains using the Kirkpatrick model, including Hanna and Semple (44) on healthcare professionals’ self-efficacy. In the context of disaster training, Cao et al. (47) demonstrated that TTX significantly improved CDC personnel’s mastery of a malaria elimination strategy, outperforming traditional teaching.

Beyond the quantitative gains reflected in NDEKS scores, our qualitative findings provided deeper insight into how this knowledge acquisition occurred. As identified in Theme 1, TTX promoted the development of a structured disaster knowledge system. Participants described moving from fragmented understanding to integrated frameworks, with one nurse noting: “After the competition program, I reviewed the entire knowledge framework and documented it in a memo.” This aligns with Silenas et al. (48), who found that role-playing in TTX helps participants integrate disparate information into systematic frameworks, transforming isolated facts into coherent, applicable knowledge structures.

The most pronounced quantitative improvement in our study was in “triage” (*Z* = 7.464, *p* < 0.001). This may be attributed to the fact that triage is a core component of disaster response, and TTX’s scenario-based design provides repeated practice opportunities for nurses to apply triage protocols in simulated emergency situations (42, 43). In contrast, “biological preparedness” had the least apparent quantitative improvement, with a median score remaining at 3.00, though the difference was still statistically significant (*Z* = 5.281, *p* < 0.001). This aligns with the results of Lin et al. (49), who noted that nurses often lack hands-on experience in specialized areas such as biological hazard response in daily clinical work, making it more difficult to improve through short-term TTX training alone.

#### 4.2.3 Level 3 (behavior): fostering core foundational capacities for practice change

Level 3 evaluates the extent to which participants apply their new knowledge and skills in the workplace. While a direct, longitudinal observation of behavior change was beyond the scope of this immediate post-training study, we used the EPQ as a proximal indicator. This scale measures participants’ perceptions of educational practices—such as active learning, collaboration, and high performance expectations—which are well-established precursors to subsequent behavior change. The high EPQ scores (overall 4.40 ± 0.55), with collaboration rated highest, indicate that the TTX program successfully fostered an environment conducive to translating learning into clinical practice. This finding is echoed in qualitative research by Brunero et al. (50), who found that TTX for violence prevention helped health practitioners achieve “role clarity,” a key component of effective workplace behavior. Our qualitative findings (Theme 2: enhancing critical thinking, teamwork, and communication skills) further support this, with nurses reporting that the collaborative problem-solving in the TTX would directly influence their future professional actions. This approach to evaluating Level 3 using participant perceptions of enabling factors is methodologically sound and consistent with practices in other healthcare education studies (22, 27), which often use self-reported confidence and perceived utility as indicators of potential behavioral application.

#### 4.2.4 Level 4 (results): evidence of organizational and professional impact

The highest level, Level 4, measures the training’s long-term, broad-scale results, such as improved organizational outcomes or patient care. Our survey of the 30 participating healthcare institutions provided tangible evidence of the TTX program’s impact at this level. The extensive disaster nursing activities reported for 2025—including 15 emergency drills, the establishment of new municipal disaster nursing committees, and widespread public education campaigns—suggest that the TTX program has not only equipped individual nurses with skills but has also catalyzed systemic change and capacity building across the province. This “ripple effect” is a hallmark of a successful training program.

The multidisciplinary collaboration experienced by participants during the TTX, as highlighted in our qualitative findings (Theme 4: strengthening multidisciplinary collaboration), may serve as a foundational enabler for these broader organizational outcomes. Nurses reported that the TTX experience enhanced their understanding of multidisciplinary knowledge and enabled them to analyze problems from different disciplinary perspectives, with one participant noting it was “similar to a multidisciplinary case discussion.” This heightened appreciation for collaborative practice, cultivated during the simulation, likely contributed to participants’ ability to initiate and engage in the wide range of disaster nursing activities observed at the institutional level. Building on the momentum generated by the program, participating nurses became actively involved in academic activities, including contributing to the development of a forthcoming disaster nursing textbook, *Emergency Medical Rescue Nurse Training Manual* (in press, People’s Medical Publishing House). Together with the establishment of new municipal committees and the widespread public education campaigns, these efforts represent tangible results at the organizational level, demonstrating that the program’s impact extended beyond individual skill development to systemic capacity building across the province.

### 4.3 Comparing TTX with other training modalities: toward an integrated disaster training framework

In disaster training for healthcare professionals, TTX, FSE, and HFS—which encompasses virtual reality, augmented reality, and mixed reality technologies—each offer distinct yet complementary strengths; their combined use holds great promise for enhancing the comprehensiveness of training (51). FSE excels in replicating real-world disaster conditions, providing full-scale, hands-on experience that hones team coordination, resource allocation, and stress management in lifelike scenarios, making it invaluable for validating organizational emergency response systems (52). HFS stands out for its immersive, controlled simulations, effectively boosting learner motivation, refining complex practical skills like triage accuracy, and allowing repeated practice of high-pressure scenarios without real-world risks (51, 53). More recently, augmented reality and mixed reality have emerged as complementary modalities, blending digital elements with the physical environment to create contextually rich learning experiences (51). While virtual reality creates a fully simulated environment, augmented reality overlays computer-generated imagery onto the real world, and mixed reality allows users to interact with both physical and virtual objects simultaneously, offering further opportunities for enhancing disaster response training (51). TTX, while more straightforward in format, is equally effective in fostering role clarity, interprofessional collaboration, and structured knowledge application, with the added advantages of low cost, minimal resource requirements, and accessibility in resource-constrained settings (30, 50).

For instance, a quasi-experimental study comparing TTX and FSE among paramedic students found both significantly improved disaster preparedness, with TTX achieving slightly higher scores in learning outcomes, trust, and perceived usefulness (18), aligning with findings from nursing-focused research where TTX was highly rated for enhancing collaboration and knowledge retention. Similarly, HFS was shown to outperform traditional methods in learner engagement and triage accuracy (20), yet its high hardware and technical costs contrast with TTX’s cost-efficiency, as demonstrated by Sena et al. (43) who noted TTX’s feasibility for widespread implementation across healthcare institutions. A scoping review by Frégeau et al. (19) further highlighted TTX’s value in strengthening interprofessional collaboration and policy familiarity, complementing HFS’s strengths in high-fidelity immersion and FSE’s focus on system-level readiness. Future training models could create synergy by integrating TTX to establish foundational frameworks, HFS to provide an immersive environment, and FSE to validate full-system responsiveness, thereby maximizing each method’s unique benefits.

### 4.4 Limitations

This study has several limitations. First, the sample was limited to nurses from Anhui Province, China, which may limit the generalizability of findings to other regions or healthcare systems. Future research should include participants from diverse geographic and institutional backgrounds to enhance external validity. Second, while we employed the Kirkpatrick model as a robust framework, our assessment of Level 3 (Behavior) relied on a proximal indicator (the EPQ) rather than direct observation of practice change. Future studies should incorporate longitudinal designs with direct observation, supervisor ratings, or self-reported behavior change at 3–6 months post-training to more definitively capture skill application in real-world settings. Third, although we documented significant organizational activities at Level 4 (Results), attributing these solely to the TTX program is challenging, as they may be influenced by concurrent initiatives. Finally, while we compared TTX with other modalities through literature review, direct comparative research (e.g., TTX vs. HFS vs. FSE) is needed to quantify their relative effectiveness in different training contexts.

Future studies should also explore the integration of TTX with emerging technologies (e.g., virtual reality, augmented reality, and mixed reality) to enhance realism and develop standardized TTX protocols for different disaster types. Additionally, qualitative research on participants’ long-term experiences and the impact of TTX training on actual disaster response outcomes will provide valuable insights for optimizing training programs.

## 5 Conclusion

This study demonstrates that a competitive TTX program, evaluated through Kirkpatrick’s Four-Level Model, is a scientific and effective training approach that enhances nurses’ mastery of disaster rescue knowledge, sharpens their critical thinking, teamwork, and communication skills, strengthens interdisciplinary collaboration, and provides a solid foundation for subsequent practical simulation training. Within the diverse landscape of disaster training methods, TTX occupies a unique position due to its balance of effectiveness, cost-efficiency, and operability. While other training approaches may excel in specific aspects such as technical advancement or scenario realism, TTX’s accessibility and focus on collaborative problem-solving make it a valuable component of disaster nursing training.

## Data Availability

The raw data supporting the conclusions of this article will be made available by the authors, without undue reservation.
