# Supplementary material for: Disaster preparedness knowledge and experiences among nurses during a competitive tabletop exercise program
Source: Front Public Health. 2026 Apr 24;14:1774598. doi: 10.3389/fpubh.2026.1774598 (PMC13153131; doi:10.3389/fpubh.2026.1774598)
Supplement: Supplementary file 1 [file Data_Sheet_1.PDF]

Supplementary Appendix 1. List of the 30 participating healthcare institutions

1. The First Affiliated Hospital of Anhui Medical University
2. The Second Affiliated Hospital of Anhui Medical University
3. First Affiliated Hospital of University of Science and Technology of China (Anhui Provincial Hospital)
4. Anhui Public Health Clinical Center
5. The First Affiliated Hospital of Anhui University of Traditional Chinese Medicine
6. The Second Affiliated Hospital of Anhui University of Traditional Chinese Medicine
7. The First Affiliated Hospital of Anhui University of Science and Technology
8. The First Affiliated Hospital of Bengbu Medical University
9. The Second Affiliated Hospital of Bengbu Medical University
10. Wannan Medical University Affiliated Yijishan Hospital
11. The Second Affiliated Hospital of Wannan Medical College
12. The 901st Hospital of the Joint Logistics and Security Force of the Chinese People's Liberation Army
13. Anhui No.2 Provincial People's Hospital
14. Hefei First People's Hospital
15. Anhui Armed Police Corps Hospital
16. Chaohu Hospital Affiliated to Anhui Medical University
17. Fuyang Hospital Affiliated to Anhui Medical University
18. Lu'an Hospital Affiliated to Anhui Medical University
19. Anqing First People's Hospital of Anhui Province
20. The Third People's Hospital of Bengbu
21. Bozhou People's Hospital
22. The People's Hospital of Chizhou
23. The Second People's Hospital of Fuyang City
24. Huangshan People's Hospital
25. Maanshan People's Hospital
26. Tongling People's Hospital
27. The Fourth People's Hospital of Wuhu City
28. Suzhou Municipal Hospital
29. Xuancheng People's Hospital
30. Naval Anqing Hospital
