# Supplementary material for: Disaster preparedness knowledge and experiences among nurses during a competitive tabletop exercise program
Source: Front Public Health. 2026 Apr 24;14:1774598. doi: 10.3389/fpubh.2026.1774598 (PMC13153131; doi:10.3389/fpubh.2026.1774598)
Supplement: Supplementary file 2 [file Data_Sheet_2.PDF]

Supplementary Appendix 2. The CO- S- TR model and explanation of 12 Elements

The CO- S- TR model is a standardized framework for surge capacity assessment and mass casualty incident (MCI) response, integrating incident management, logistical support, and casualty care functions [1,2,3]. It is widely applied in hospital emergency preparedness, tabletop exercises (TTX), and incident command system (ICS) implementation [2,3].

1. C4: Command, Control, Communication, Coordination

Command: Establish a unified incident command system and designate an incident commander.
Control: Ensure on-site safety, situation assessment, and dynamic incident management.
Communication: Maintain stable internal and external information transmission.
Coordination: Collaborate across departments, hospitals, and emergency agencies.

2. S4: Staff, Stuff, Space, Special

Staff: Mobilize and deploy medical and support personnel.
Stuff: Allocate medical supplies, equipment, and logistical resources.
Space: Set up triage, treatment, and temporary surge areas.
Special: Address contamination, security, high-risk conditions, and special population needs.

3. T4: Tracking, Triage, Treatment, Transport

Tracking: Record and monitor casualty information and disposition in real time.
Triage: Rapidly classify patients by injury severity to prioritize care.
Treatment: Provide evidence-based, priority-driven emergency care.
Transport: Arrange safe, coordinated inter- and intra-hospital patient transfer.

TABLE S1. The CO- S- TR Framework

**The CO- S- TR Framework**		
**C4**	**S4**	**T4**
Command	Staff	Tracking
Control	Stuff	Triage
Communication	Space	Treatment
Coordination	Special	Transport

References for Appendix 2

1. Hick JL, Koenig KL, Barbisch D, Bey TA. Surge capacity concepts for health care

facilities: the CO-S-TR model for initial incident assessment. *Disaster Med Public Health Prep*. (2008) 2 Suppl 1:S51-7. doi:10.1097/DMP.0b013e31817fffe8
2. Liu J, Huang Y, Li B, Gui L, Zhou L. Development and Evaluation of Innovative and Practical Table-top Exercises Based on a Real Mass-Casualty Incident. *Disaster Med Public Health Prep*. (2022) 17:e200. doi:10.1017/dmp.2022.95
3. Rajapaksha NU, Abeysena C, Balasuriya A, Wijesinghe MSD, Manilgama S, Alemu YA. Incidence management system of the healthcare institutions for disaster management in Sri Lanka. *BMC Emerg Med*. (2023) 23:6. doi:10.1186/s12873-023-00777-y
