# Supplementary material for: Disaster preparedness knowledge and experiences among nurses during a competitive tabletop exercise program
Source: Front Public Health. 2026 Apr 24;14:1774598. doi: 10.3389/fpubh.2026.1774598 (PMC13153131; doi:10.3389/fpubh.2026.1774598)
Supplement: Supplementary file 3 [file Data_Sheet_3.PDF]

Supplementary Appendix 3. Detailed descriptions of the six domains of the Nursing Disaster Emergency Knowledge Scale (NDEKS)

Based on the NDEKS instrument, the six domains assess the following knowledge areas:

1. Incident command system in disaster care: (1) Disaster command system responsibility assignment, (2) Disaster response capability, (3) Nursing plan, (4) Function of command system, (5) Safety evaluation, (6) The difference in incident command between emergency and non-emergency situations, (7) Volunteer duties.

2. Triage: (1) Quick physical assessment of the injured, (2) Quick psychological assessment of the injured, (3) Assisted inspection classification, (4) Basic first aid and technology, (5) Assessment of effectiveness of measures.

3. Communication: (1) Disaster nursing records, (2) On-site organization and management, (3) Transportation and critically ill patient information communication procedures, (4) Providing disaster avoidance and mitigation safety information, (5) Assisting and cooperating with emergency surgery capabilities, (6) Post-disaster analysis summary, (7) Lifeline support system assessment.

4. Special care, isolation and decontamination: (1) Post-traumatic stress in children and adolescents, (2) Provision of psychological support, (3) Care for sensitive and vulnerable groups, (4) Publicity and education in response to disasters such as biological agents, nuclear, chemical, and explosion incidents, (5) Mental health protection for rescuers, (6) Common psychological crisis identification, (7) Isolation procedures for human exposure to biological or chemical agents, (8) Selection of appropriate personal protective equipment when caring for patients exposed to biological, chemical, or radioactive agents, (9) Hospital and community quarantine procedures, (10) Decontamination procedures for out-of-hospital rescue operations, (11) Nuclear, biological and chemical decontamination, (12) Familiarity with specific biochemical detoxification and preventive medications, (13) Identification of exposure to infectious diseases based on symptoms and medical history, (14) Key points of rescue teams in the early, middle and late stages, (15) Principles of corpse treatment.

5. Reporting and access to essential resources: (1) Understanding of diseases that must be reported to the National Health Commission immediately, (2) Symptoms that need to be reported to a doctor or expert immediately, (3) Communication with relevant departments and agencies about typical or special cases.

6. Biological preparedness: (1) Precautions for vaccine injection, (2) Transmission characteristics of different microbial diseases, (3) Identification of special microbial infection symptoms.
